# Current state of poultry waste management practices in Bangladesh, environmental concerns, and future recommendations

**DOI:** 10.5455/javar.2022.i618

**Published:** 2022-09-30

**Authors:** Md. Masudur Rahman, Alamgir Hassan, Ismail Hossain, Mohammad Mofizur Rahman Jahangir, Emdadul Haque Chowdhury, Rokshana Parvin

**Affiliations:** 1 Nourish Poultry and Hatchery Ltd., Dhaka, Bangladesh; 2 Department of Pathology, Faculty of Veterinary Science, Bangladesh Agricultural University, Mymensingh, Bangladesh; 3 Department of Soil Science, Faculty of Agriculture, Bangladesh Agricultural University, Mymensingh, Bangladesh

**Keywords:** Poultry waste, waste generation and utilization in Bangladesh, environmental impact, modern technology, waste mitigation

## Abstract

This review paper focuses on the current state of poultry waste generation, composition, and management techniques in commercial poultry farms and trading in Bangladesh, to reduce pollution and generate economic benefits from poultry waste. It also underlines the negative impact of poultry waste disposal on the environment. In Bangladesh, collection of poultry waste into bags and, subsequently, direct use as fertilizer in agricultural fields and aquaculture is common, while alternative disposal methods such as composting and biogas generation are now attracting commercial poultry producers. Direct use of poultry manure results in poor air and soil quality, environmental deterioration, detrimental effects on global health, climate change due to high levels of atmospheric ammonia, and the creation of significant amounts of greenhouse gases. Lack of knowledge and investment, as well as high demand for free land for composting, are important obstacles. Future research on precise waste characterization, improved understanding of poultry waste management, and increased efforts on developed waste disposal for a safe environment are therefore recommended. So, poultry waste, which is currently a burden for the environment, could be turned into a useful agricultural resource, which would be useful for the poultry industry.

## Introduction

Poultry is an important subsector of the livestock industry and the fastest-growing agricultural enterprise in Bangladesh. It contributes significantly to the economy of Bangladesh by providing employment opportunities in rural and semiurban areas, as well as accessible protein sources for the growing population [1]. Poultry production has grown from 91 million in 1,990 to 365.85 million in the fiscal year of 2020–2021, owing to the huge demand for poultry meat and eggs [2,3].

Bangladesh has a long history of poultry production in traditional backyard farming. Initially, poor rural women and unemployed youngsters were involved in commercial poultry rearing on a modest scale. It also included semiurban and urban poultry raisers who contributed to meeting the growing demand for eggs and meat. Since 1990, the commercial poultry sector has grown at a steady pace of 15%–20% per year, with a slight slowdown after 2007 due to the avian influenza outbreak by Hamid et al.[4], contributing about 1% of the country’s gross domestic product (GDP) [1]. However, the overall investment in the poultry business in 2019 was around 3.7 billion Bangladeshi taka or 42 million USD (Larive International B.V. and Light Castle Partners Ltd., 2020), and commercial poultry production was carried out on around 150,000 farms [1].

Poultry production, on the other hand, is a complicated process that combines the economic, social, and environmental aspects of a given product within a socioeconomic context. Unfortunately, as poultry production has become more concentrated and operation sizes have expanded, major environmental challenges have arisen [5]. Despite their great socioeconomic benefits in terms of egg production, meat production, and employment generation, they also endanger human and animal lives by polluting water, land, and air[6–8].

The primary issue facing the poultry industry is the large-scale accumulation of wastes [9] from hatcheries and slaughterhouses (water and organic solid by-products) of live bird markets (LBM). Poor waste management can cause health and welfare problems in flocks, bad smells, fly breeding, and contamination of land and water [10,11].

As a result, poultry waste management is essential for environmental protection, human health and safety, and safe poultry production. Such a significant volume of waste may cause disposal and pollution difficulties unless environmentally and economically sustainable alternatives are discovered. Resolving these issues requires serious attention to managing poultry waste so that the environmental impacts of such a huge volume of waste are minimized.

Therefore, this review focuses on the current state of the commercial poultry waste management practices in Bangladesh, emphasizing their significance as well as proposing a successful guideline to manage the waste properly. Researchers or scientists will quickly understand what is happening, which will help improve waste management and a recycling system that will last.

## Characterization and Quantification of Poultry Waste

Poultry wastes or manure consist of excreta, feathers, spilled feeds and water, dead birds, broken eggs, wastewater, litter or bedding materials (i.e., rice husk, sawdust, wood shavings, peanut hulls, straw, etc.), dead birds, and slaughterhouse and hatchery wastes. The primary components of poultry wastes are water and carbon; small amounts of nitrogen and phosphorous; and trace amounts of chlorine, calcium, magnesium, sodium, manganese, iron, copper, zinc, potassium, arsenic, cobalt, selenium, molybdenum, and boron [9,12,13].

According to previous research, offal (slaughterhouse waste) contains 5.3% of the total Kjeldahl nitrogen, 32% protein, 54% lipids, and 0.6%–0.9% methane (CH_4_) generation potential, whereas chicken feathers contain approximately 91% protein (mostly keratin), 1% lipids, and 8% water [8]. The detailed chemical composition of poultry and hatchery waste is shown in Table 1.

According to the Agricultural Census 2008, there was about 137.23 million poultry in Bangladesh. The total annual fresh poultry litter production was 4.52 million tons, of which 3.10 million tons were produced under commercial poultry farming [21]. A study argued that Bangladesh produces 1.56 million tons of poultry manure annually [22]. According to the International Finance Corporation, around 2,000 million chickens in Bangladesh produce about 2.2 million tons of manure annually [23]. Another recent study found that the country’s 312 million poultry produce 2.1 million tons of poultry waste (dry matter) yearly [24]. Specifically, a layer flock of 3,000 birds produces 300 kg of feces per day while consuming 500 kg of feed, resulting in 0.1 kg/bird/day waste generation. Another study that claimed a flock of 10,000 laying hens would produce one metric ton of waste daily supports the above findings [25,26]. On average, on a farm, commercial layer, broiler, or breeder poultry excretes 0.07–0.178 kg of feces per day. However, the feces production range indicates a lot of fluctuation due to differences in feed intake, water consumption, environment, bird age, composition, and feed form[17,19,27–29].

**Table 1.** Chemical composition of poultry and hatchery wastes.

Particulars	Poultry waste	Hatchery waste	Source
Dry matter %	N/A	94.66–97.35	[14–20]
pH	6	N/A	
Metabolizable energy (Kcal/kg)	N/A	2,914	
Crude protein %	N/A	22.80–44.58	
Crude fiber %	N/A	0.58–8.06	
Ether extract %	N/A	11.43–30.00	
Nitrogen-free extract %	N/A	21.31–23.98	
Ash %	N/A	14.00–40.00	
Organic carbon%	35.4	N/A	
Nitrogen %	3.52	N/A	
Phosphorus %	0.86	0.39–0.84	
Potassium %	1.83	N/A	
Calcium %	0.45–2.05	7.26–22.60	
Magnesium %	0.215–0.51	N/A	
Iron (ppm)	950–1050	N/A	
Manganese (ppm)	80–240	N/A	
Zinc (ppm)	70–230	N/A	
Copper (ppm)	12–60.4	N/A	

Note: N/A, Information not available

## Usage of Poultry Waste in Bangladesh

Feathers, offal, and litter from poultry are valuable agricultural wastes because they contain nutrients and are a rich source of protein. They can be made into valuable products like feather meal, biodiesel, biodegradable plastic, fertilizer, or manure [13].

### Application as fertilizer

Poultry litter is an excellent organic fertilizer for enhancing soil fertility, root system development, and plant vigor, as well as making the plant more resistant to diseases and pest infestations [30,31]. Direct application of poultry waste in crop fields, on the other hand, can cause soil, air, and water quality concerns that are yet to be investigated. Most of the poultry manure and litter are currently applied to agricultural land, making it the preferred and practical method in developing nations [32,33]. Surface and groundwater become polluted because of the potential pollutants in the manure and litter, and agricultural yields drop when there is a lot of manure and trace elements [13].

### Composting

Composting, an anaerobic conversion process that turns organic compounds into a soil-friendly product in a controlled manner, is one of the most successful and widely used methods for dealing with poultry waste [13,34,35]. Composting has several advantages, including the slow release of nutrients, lower pathogen levels, less potential to degrade water quality, fewer weed seeds, improved soil fertility, reduced fly infestations, and foul odor [21,34,36]. Composting, on the other hand, necessitates land, financial investment, labor, and management and emits harmful CH_4_ gas as it decomposes [34,35,37]. However, vermicompost could be a better option that minimizes the environmental effect. Vermicompost is created by decomposing poultry waste and bedding materials by various worms, typically red wigglers, white worms, and other earthworms (https://www.adrabangladesh.org/). It is currently gaining popularity due to its superior quality for the soil, plants, and vegetable fields. Nowadays, private companies in Bangladesh are currently marketing vermicompost at a low cost to farmers (https://www.garden.com.bd/fertilizers/2-vermicompost-fertilizer.html).

Moreover, government and nongovernment organizations (NGOs), as well as research and extension institutes, such as Bangladesh Agricultural University and Bangladesh Agriculture Research Institute, are bridging farmers’ access to the most appropriate and cost-effective composting technologies [37]. Private organizations, such as Waste Concern, Annapurna Agro Service, Grameen Shakti, Rural Development Academy, Innovation Consulting Pvt. Ltd., Rash Agro Enterprise, and Acme Laboratories, have evolved to work toward the commercial availability of compost in superstores in Bangladesh and the promotion of composting [37]. These organizations assist rural farmers in preparing compost by regulatory guidelines. Although the support is minimal, the capacity of waste supply for these private enterprises is insufficient. Wood ash, tea leaves, table scraps, sawdust, leaves, fruit and vegetable scraps, garden weeds, eggshells, and chicken manure are all used in these businesses. So, in those businesses, only a small amount of chicken waste is used to make compost.

### Biogas

Biogas is a gas mixture formed by the anaerobic digestion of various organic materials. It is largely composed of CH_4_, carbon dioxide (CO_2_), and a trace amount of hydrogen sulfide [38,39]. Poultry wastes, leftover feeds, and wastewater from the premises can all be utilized to form a slurry (biomass) that can be supplied into a biogas plant for anaerobic fermentation to produce biogas [40,41]. Biogas and bio-slurry replace the cost of fuel (cooking, power, or both) and fertilizer [29,42]. Moreover, biogas plants reduce carbon emissions [29]. Bangladesh has a significant potential for producing biogas from poultry wastes as it can build at least 4 million biogas plants, which could produce 105 billion cubic feet of biogas per year, equivalent to 1.5 million tons of kerosene or 3.08 million tons of coal, and could meet the cooking and lighting needs of approximately 20% of the country’s households [43,44]. Approximately 91,350 home and commercial so-called biogas plants that use cattle and poultry manure as raw materials are currently available [24] in the country. Still, the energy production level from those biogas plants is unknown. Individual family biogas plants are currently available in Bangladesh for small- and medium-scale poultry producers, while commercial biogas plants are not yet well structured. Although biogas generated in anaerobic systems is useful for household and agricultural use, bio-slurry is bad for farmers and the environment [44]. Limestone is an essential ingredient of layer feeds, part of which usually passes through the feces and accumulates in slurry. The biogas plant’s digester gradually fills up with slurry-borne limestone, which also clogs the plant’s slurry drainage system, causing producers to stop operating. On the other hand, with regular use, the biogas plant becomes so dirty and smelly that it provokes complaints of odor from neighbors. These prevent Bangladesh from fully utilizing the advantages of its biogas plant (personal observation).

### Biochar production

Using biochar, a solid formed from carbonized biomass, on land can enhance soil properties and lower greenhouse gas emissions brought on by biomass degradation [45]. In subtropical climates, poultry waste biochar can reduce nitrogen mineralization rates, resulting in higher nitrogen usage efficiency and crop yield [46,47]. It is made by pyrolyzing or gasifying biomass feedstock in an oxygen-depleted environment to create a stable, carbon-rich product resistant to soil deterioration. Poultry litter biochar is made from fresh poultry litter by pyrolyzing it for 10 min at 300°C in a muffle furnace with low oxygen. It can also be produced on a large scale using a simple biochar plant [48]. It contains higher nutrients than fresh poultry litter and hence is used on agricultural land as a long-term production management technique [45,47]. However, in Bangladesh, so far, the method is not being used to dispose of poultry waste. Also, the process is sensitive to changes in temperature, gas levels, pH, ash concentration, and tar content. This makes it hard to use biochar waste treatment methods.

### Livestock feeds

Processed poultry litter is used in diets for poultry, swine, lambs, ewes, lactating cows, wintering cattle, and brood cows [49–51]. Feathers contain more than 90% protein and are a good source of hydrophobic amino acids like cysteine, arginine, and threonine; feathers are also processed into feather meal for animal feed, organic fertilizer, and feed supplements [8]. There is currently no feed company in Bangladesh that uses poultry litter as an ingredient in livestock feed.

### Rendering (poultry meat and by-product processing)

Rendering is a process of both physical and chemical transformation that involves the use of a variety of equipment and processes, including the application of heat, de-moisturizing, and the separation of fat[52].Rendering transforms by-products of meat and poultry into marketable commodities such as edible and inedible fats and proteins for agricultural and industrial applications [53,54].It can produce meat-bone-meal, poultry by-product meal, and steam hydrolyzed feather meal for fish or livestock feed or used as fertilizer by further processing via anaerobic digestion or composting [55]. The materials used in the rendering process included viscera, meat scraps (including fat, bone, and blood), feathers, hatchery by-products (infertile eggs and dead embryos), and dead animals [53]. The rendering process is also not yet commercialized in Bangladesh.

In addition, there are many other uses of poultry waste, like preparation of biodiesel, reuse of litter, and mass burning or direct combustion. Feather meal is boiled in water at 70°C to remove the fat, which is then trans-esterified to produce biodiesel [8]. Reusing litter materials for broiler rearing is a popular idea in Bangladesh due to the cost-saving advantages of not completely replacing the leftover litter with a fresh bedding material for each batch [8]. Direct burning and incineration are well-known ways of generating renewable energy and ash from waste, and they have the potential to close the nutrient loop in the poultry industry [56]. None of these uses are being practiced in Bangladesh.

#### Present Scenario of Waste Management in Bangladesh

There are approximately 8 grandparent stocks, 100 breeder farms and hatcheries, and over 70,000 commercial layer and broiler farms in Bangladesh [57], producing 2.1 million tons of stinky manure each year [24], which provokes complaints of odor and flies from neighborhoods. The demand for manure in the country is seasonal and most of it is piled up for the rest of the year.

### Farm waste

**Figure 1.**
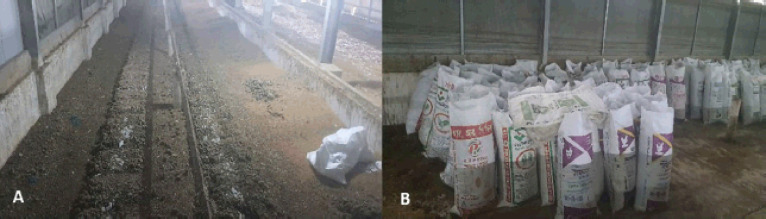
Poultry litter management in a slat cum floor system of a breeder farm in Bangladesh. A) Poultry excreta under the slat area for a period of 66 weeks. B) Collected excreta from the shed for sale.

**Figure 2.**
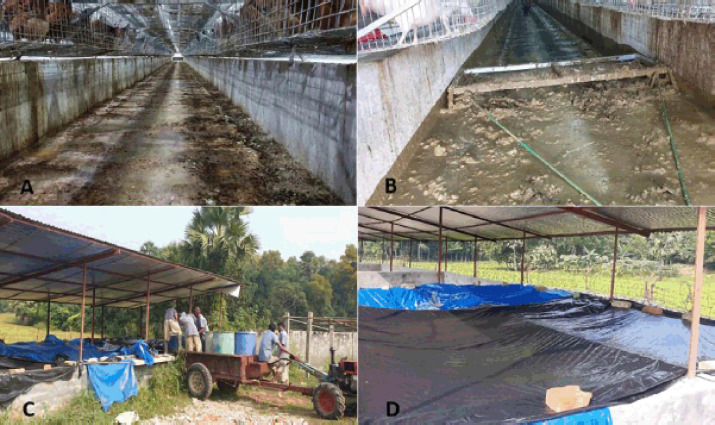
Poultry litter management in case of breeder or commercial layer farms in Bangladesh. A) and B) Litter for scrubbing out from the shed. C) Compost chamber. D) Anaerobic condition for preparing compost.

**Figure 3.**
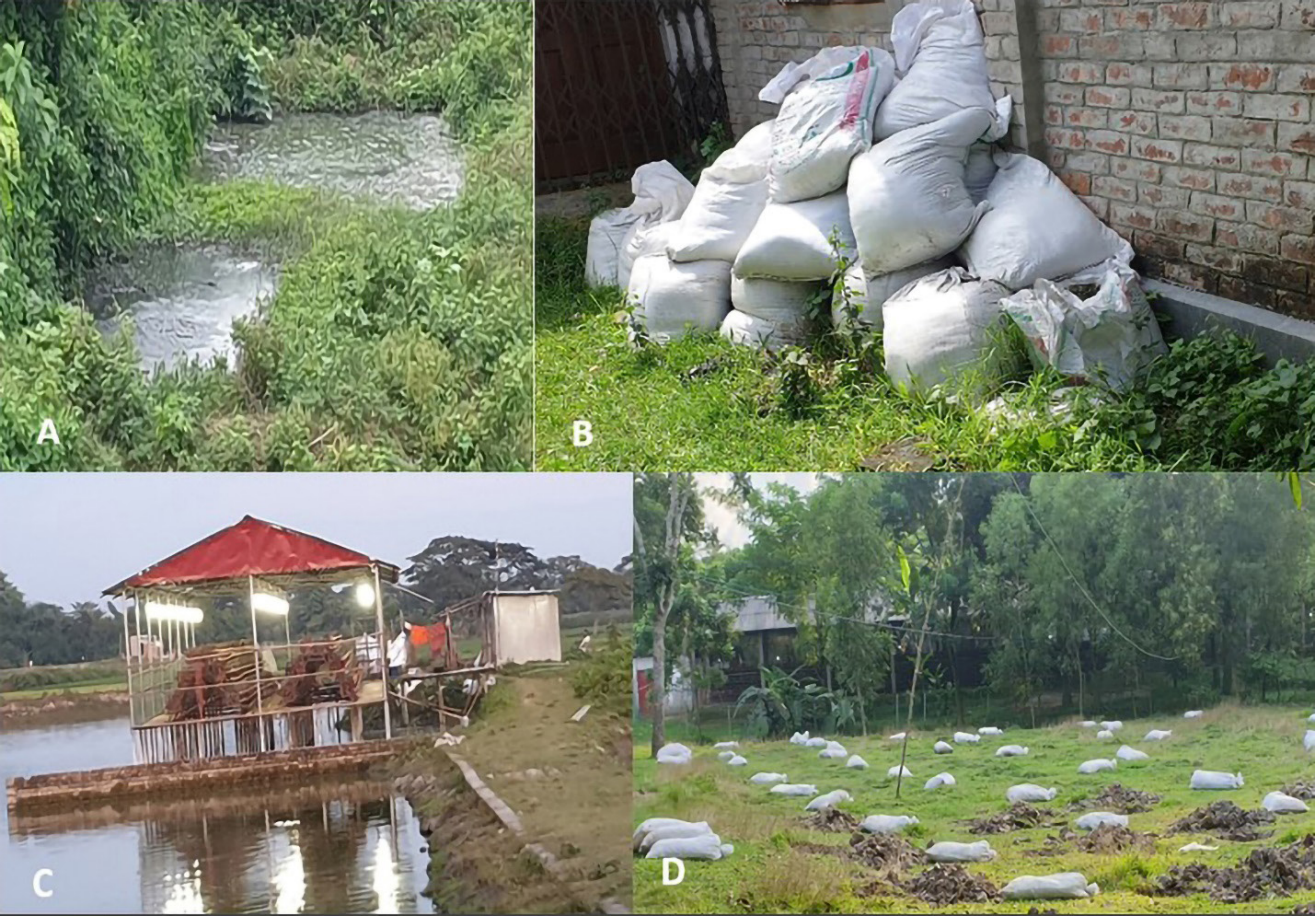
Traditional waste management practices in commercial layer and broiler farms in Bangladesh. A) Waste passing directly to the open pit. B) Before being sold, the litter is collected and stored beside the shed. C) Integrated layer and fish farm where the litter directly goes to the pond. D) Litter used in a nearby agricultural land.

Generally, the large poultry breeder companies, such as Aftab Bahumukhi Farms Ltd., Nourish Poultry and Hatchery Ltd., Kazi Farms Group, Nahar Agro Ltd., Provita, Paragon Group, and others, have strong relationships with local brokers who collect spent litter and sell it to crop and fish farmers. These companies manage their poultry in contact housing, either in floor or slat cum floor systems, or in cage systems, with birds kept for 64–66 weeks (broiler breeders), 72–80 weeks (layer breeders), and approximately 90 weeks (layers). They usually practice proper poultry waste management, primarily through composting (Fig. 1). The litter and/or manure remain in the shed until the birds are culled. In a cage system, farmers routinely scrub the litter and keep it in a compost pit (Fig. 2), while some farmers store it in an open field. Small-scale commercial layer and broiler farms have haphazard poultry waste management, with the waste being utilized as fertilizer on neighboring agricultural land, piled at the roadside or near the shed, sold to the buyer, used as fish feed, or dumped in open areas (Fig. 3) without any treatment [3,27,58–60]. Due to economic effectiveness, farmers in the broiler production system frequently reuse their litter materials (rice husk) for two or more flocks, which result in poor quality poultry manure if not adequately dried, increasing the risk of disease outbreaks in that flock. In addition, litter from rural poultry farms is commonly deposited in nearby low-lying areas, resulting in greenhouse gas emissions as well as pollution of the water and air [25]. The majority of Bangladeshi poultry farms use litter as compost (52.78%), while others use it for fish feed (30.55%) and other purposes (8.33%). About 47.20% of the farms dispose of litter in pits, while 44.44% in open places, 5.56% on the roadside, and the rest 2.78% in other places, as reported previously [61]. Furthermore, poultry waste is disposed of in agricultural fields, open disposal pits, or open drains, and poultry carcasses are routinely disposed of or thrown near poultry sheds, where dogs and foxes scavenge for food [44,59].

**Figure 4.**
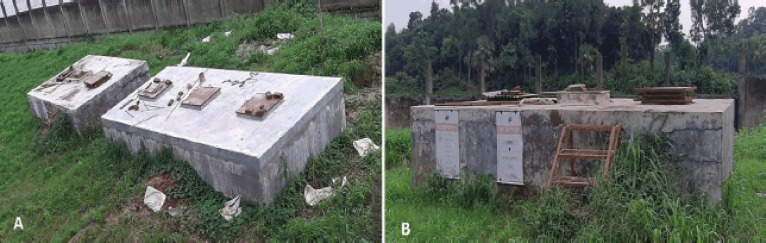
Disposal pit for dead and culled birds of a breeder farm in Bangladesh. A) Top view. B) Side view.

In most cases, large commercial companies have their own disposal pit (Fig. 4) to dispose of dead and culled birds. Some poultry companies convert manure into organic fertilizer as it is becoming popular among vegetable producers. However, biogas plants are not yet established at most private companies because these plants require a lot of land and a huge investment to build. Kazi Farms Group (one of Bangladesh’s largest corporations) had previously established biogas units. Still, they were compelled to shut down the plants due to a lack of space on their own property for the large amounts of produced slurry. The stored slurry wastes rather flooded across the public’s land during the rainy season, resulting in a slew of compensation claims, prompting the company to shut down the biogas plant and compost organic fertilizer [26]. However, those companies may retreat slurries by vermicomposting, and they can be transformed into organic fertilizer, which has a high commercial value. Now, vermicompost from the slurry is sold at 10 USD/bag (100 kg).

### Hatchery waste

**Figure 5.**
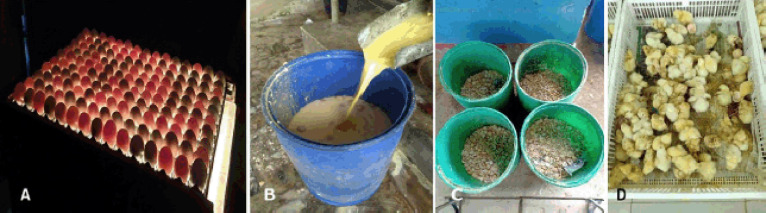
Hatchery wastes in Bangladesh. A) Candling of eggs in a hatchery that shows infertile eggs as clear eggs. B) Infertile egg fluid for dumping. C) Crushed eggshells after regular hatch out. D) Culled chicks immediately after hatch out.

The common hatchery wastes (Fig. 5) produced by the poultry hatchery are eggshells, broken eggs, infertile eggs, culled chicks, etc. Hatchery waste may be referred to as miscellaneous waste too. The traditional method of landfilling is widely used to dispose of hatchery waste throughout the country, while some companies donate embryos and culled chicks to catfish farming in ponds. Some hatcheries sell infertile eggs to bakeries for biscuits and cake preparation. Disposing of large-scale hatchery waste via landfilling is one of the key issues. As a result, the poultry sector is dealing with high land costs and a waste management problem. However, reducing hatchery waste for livestock feed production or other chemical companies (beauty soap, shampoo, conditioner, etc.) may contribute significantly to waste utilization without posing any environmental risks.

### Live bird market waste

During slaughtering and processing, retailers keep the offal and leftover chicken parts, except the feathers, and sell these as fish feed, chicken feces, feathers, and other solid chicken waste are disposed of in municipal waste disposal bins [59]. Poultry litter and leftover feed (kept at the retailer’s stall for rearing chickens before being sold) are frequently swept up, and part of these wastes adhere daily layer by layer on the surface of the floor; however, neither the market authority nor the shopkeeper care for them [59]. Other fowl parts, such as the liver, skin, and feet, are frequently sold directly to low-income community members for consumption. Because there is usually no authority to control the cleaning and disinfection method of the LBM, they are not cleaned and sanitized at the end of the day. This makes LBM the most likely place for pathogens to enter the environment and act as a hub for the spread of zoonotic avian pathogens in the community [58].

**Figure 6.**
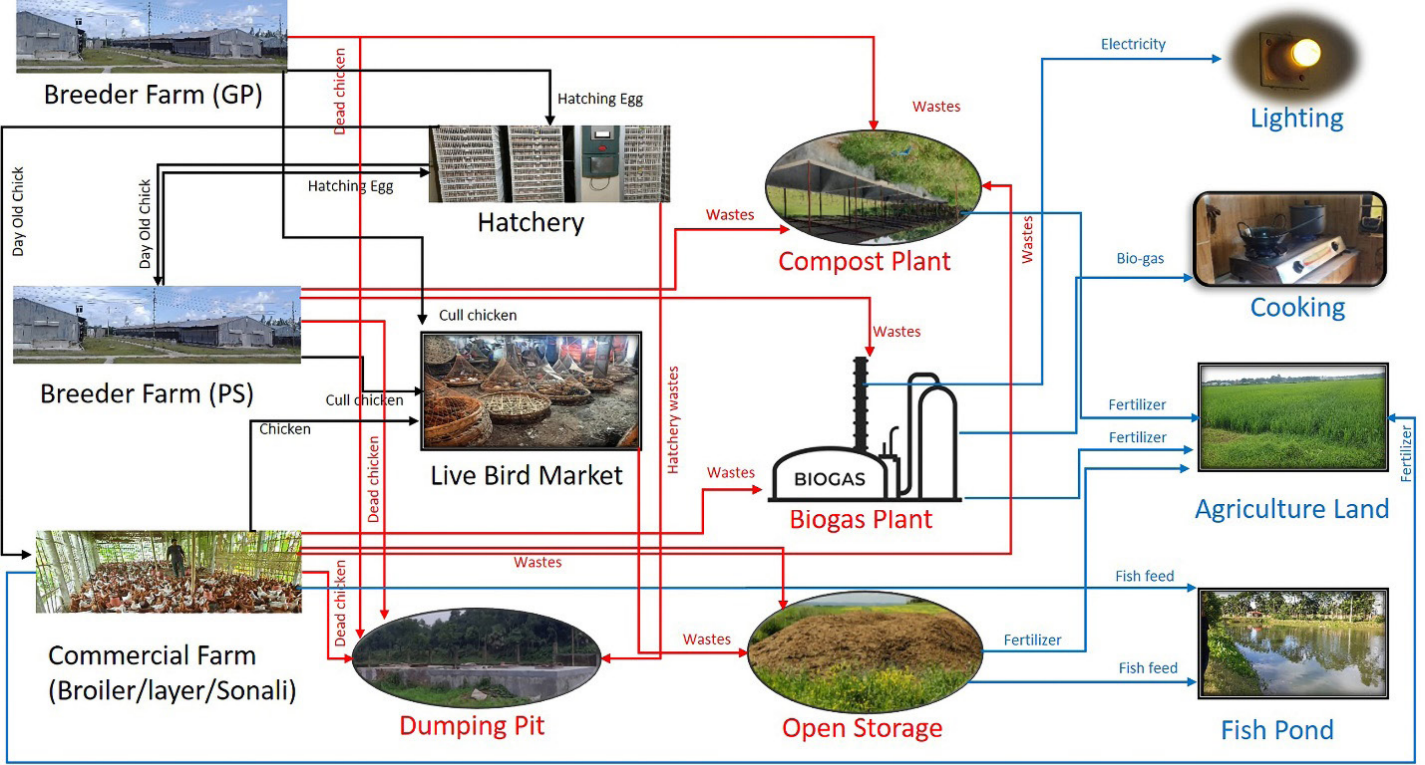
Graph illustrating the generation of poultry waste from various sources, as well as its management and utilization in Bangladesh. Black lines indicate production source; red lines indicate waste generation and management; and blue lines indicate utilization of poultry waste.

Overall, waste management practices by small- and medium-scale commercial farms are minimal, whereas large-scale producers try to maintain the scientific method. However, a lack of capital and insufficient land often impede the process. So, waste use in all types of poultry production in Bangladesh (breeder or commercial farms, hatchery, LBM) depends greatly on capital, technical knowledge, awareness, and available facilities (Fig. 6).

## Impacts on the Environment and Health

Despite the huge benefits of poultry waste, now it is becoming an undisputable concern around the world. The constant production of poultry waste causes environmental annoyances that are hazardous to animal and human health [56,62–66]. Poultry farms have been linked to poor air quality and environmental degradation due to high atmospheric ammonia emitted from poultry litter [9,67–69]. Poultry waste is contributing to global climate change by emitting greenhouse gases, such as nitrous oxide, CO_2_, and CH_4_, through microbial activity and changes in temperature, pH, moisture, and oxygen concentrations [9,70–75]. Reportedly, these air pollutants could have a significant negative impact on both human and animal health, with a variety of illnesses like nose discomfort, breathing issues, and coughing [76–78]. Long-term exposure to sustained air pollution leads to allergic reactions and effects on life span [79,80]. However, the exact number of pollutants produced from poultry waste in Bangladesh remains unknown. Many countries have found that uncontrolled disposal of poultry manure in areas where chickens are raised is a major cause of environmental damage and contamination of the air, soil, and water [56].

Poultry litter may include human and animal infections, such as zoonotic avian influenza. Therefore, appropriate hygiene must be used when handling poultry manure [80,81]. Moreover, contaminated food or water by poultry waste may contain different pathogens and water pollutants [82,83] that cause gastrointestinal diseases like typhoid fever, cholera, and hepatitis E infections [84]. Different vectors, including mosquitoes, birds, insects, and rodents, spread various diseases through waste [85,86]. Poultry waste is typically discharged through a single channel waterline that connects to ponds and rivers, causing heavy metal pollution, antibiotic residues, and microbial contamination of surface and drinking water [87–91]. Furthermore, these lead to oxygen deprivation, which could speed up the rate of toxin compound accumulation, leading to water-borne and respiratory diseases [92]. In Bangladesh, the scenario is essentially comparable, and the improper disposal of untreated poultry waste outlined above will provide an overview of the environmental impacts connected with farm operations, hatcheries, and LBM, whether directly or indirectly. Poultry farms create smells that harm the lives of individuals who live nearby. In addition, poor litter management pollutes soil and water, acts as a solid medium for pathogenic bacteria to flourish, and releases harmful compounds that result in heavy metal contamination [60,71,93,94]. It has also been demonstrated that long-term use of poultry waste on soil causes an accumulation of microelements, potentially increasing the bioavailability and toxicity of metals in the environment [87–90]. So, proper disposal, management and use are needed to reduce risks to the environment and the health of poultry.

## Conclusion and Recommendations

Scientific and safe methods of utilizing poultry waste and by-products have the potential to increase farmer revenue. In areas where intensive poultry production is practiced, and population density is high, improper poultry waste storage and disposal pose a risk of air and water pollution, as well as health hazards. Manure collection, treatment, storage, and effective utilization of poultry waste benefit the industry, the environment, and the country. The rendering process is commonly used to convert by-products into marketable products for agricultural and industrial uses [53]. The proper processing and treatment of waste material not only allows for the creation of useful and valuable by-products but also helps to save the environment. Therefore, policymakers need to consider the poultry production process and waste management in the country. For example, poultry farmers must have good facilities to dispose of their farm waste. The authority may encourage private sectors and NGOs to invest in and render the composting and biogas production processes for poultry waste or litter, LBM, and hatchery waste to render, recycle, or incinerate. The knowledge and skills of farmers can be increased through training and practice by extending services on proper poultry waste management. However, modern waste recycling, biogas plants, or composting methods require further research and implementation of the technologies at the farmer level. Government extension personnel should provide information on manure management, and successful intervention should occur through field trips, farm visits, or farmer meetings. They are highly recommended.

Perfectly managed poultry waste could be a valuable source of income and contribute to the country’s GDP, and will contribute to the United Nations Sustainable Development Goal. This is a very important problem that needs to be fixed because it is hurting the natural biological system. It should be brought to the attention of all poultry business owners and other important people.

## List of Abbreviations

GDP, Gross domestic product; LBM, Live bird market; NGO, Nongovernment organization.
